# Correlation between serum IL-32 concentration and clinical parameters of stable COPD: a retrospective clinical analysis

**DOI:** 10.1038/s41598-020-69000-3

**Published:** 2020-07-21

**Authors:** Biaoxue Rong, Tian Fu, Congxue Rong, Wen Liu, Kai Li, Hua Liu

**Affiliations:** 1 Department of Oncology and Gerontology, The First Affiliated Hospital, Xi’an Medical University, 48 Fenghao West Road, Xi’an, 710077 China; 2 School of Clinical Medicine, Xi’an Medical University, Xi’an, China; 3 Department of Respiratory Medicine, Jining NO.1 People’s Hospital, Jining, China; 4 Comprehensive Medical Department, Zhangye Second People Hospital, Zhangye, China; 5 Department of Respiratory Medicine, Minqin County People’s Hospital, Minqin, China; 6 Department of Respiratory Medicine, Gansu Provincial Hospital, Lanzhou, China

**Keywords:** Biomarkers, Diseases, Medical research, Risk factors

## Abstract

This study was to investigate the association between serum interleukin 32 (IL-32) concentration and clinical parameters in patients with stable chronic obstructive pulmonary disease (COPD). One hundred and sixteen patients with stable COPD and 70 healthy subjects were included in the study. The serum concentration of IL-32 was detected by enzyme-linked immunosorbent assay. The correlation between serum IL-32 and clinical parameters of patients with COPD was analyzed by T-test, one-way analysis of variance, multiple linear regression and receiver operating characteristic curve. The serum concentration of IL-32 in patients with stable COPD was higher than that in healthy control group (*p* < 0.001) and increased serum IL-32 was positively correlated with GOLD grading (*p* = 0.026), mMRC score (*p* = 0.004) and clinical medical history (*p* = 0.005), but negatively related to FEV1/FVC (*p* = 0.001) and FEV1% predicted (*p* = 0.001). Patient's COPD grading (*p* = 0.001), clinical medical history (*p* < 0.001) and FEV1/FVC (*p* = 0.001) exerted a significant impact on serum IL-32. The sensitivity and specificity of serum IL-32 for discerning COPD patients from healthy individuals were 85.34% and 64.29%, and the area under the curve was 0.808 (*p* < 0.001). Increased IL-32 is involved in the chronic disease progression of COPD, suggesting that IL-32 may be a molecular biomarker that reflects the severity of COPD and contributes to the disease diagnosis.

Chronic obstructive pulmonary disease (COPD), a common preventable and treatable disease, is characterized by persistent airflow limitation that is usually progressive and associated with an enhanced chronic inflammatory response in the airways and the lung to noxious particles or gases^1^. COPD is one of the diseases with high morbidity and mortality worldwide. The prevalence of COPD in Chinese population has increased year by year due to environmental pollution and population aging. Despite some efforts by the government, the disease has become a huge burden for the patient population in the country. The World Health Organization (WHO) emphasizes that early diagnosis and proper treatment are key to controlling of COPD^2^. At present, the etiology and pathogenesis of COPD are still not fully elucidated. The imbalance of various inflammatory mediators, cytokines and proteases during the development of COPD play an important role in the persistence of small airway inflammation and alveolar tissue destruction, which leads to the activation of inflammatory cell deposits and autoimmune reactions, and small airway remodeling^3–6^. Scholars generally believe that airway inflammation and immune mechanisms, protease-antiprotease imbalance, oxidative stress, autonomic dysfunction, malnutrition and climate change are involved in occurrence and development of COPD^1,4,7^.

Interleukin-32 (IL-32) is an inflammatory cytokine that can be produced by activated T cells, natural killer cells, monocytes, and epithelial cells^8,9^. IL-32 gene is located on human chromosome 16p13.3 and contains eight exons, consisting of 705 pairs of bases^10^. Previous study shows that IL-32 is highly expressed in the lung tissue of patients with COPD, and alveolar wall and bronchial epithelial cells are the main expression sites^11^, it has been confirmed to participate in the inflammatory process of COPD as a pro-inflammatory factor^12^. In this study, we examined the concentration of serum IL-32 in patients with stable COPD and explored the correlation between serum IL-32 and clinical parameters of stable COPD.

## Results

### Serum IL-32 concentration in patients with stable COPD is higher than that in healthy individuals

**Figure 1:**
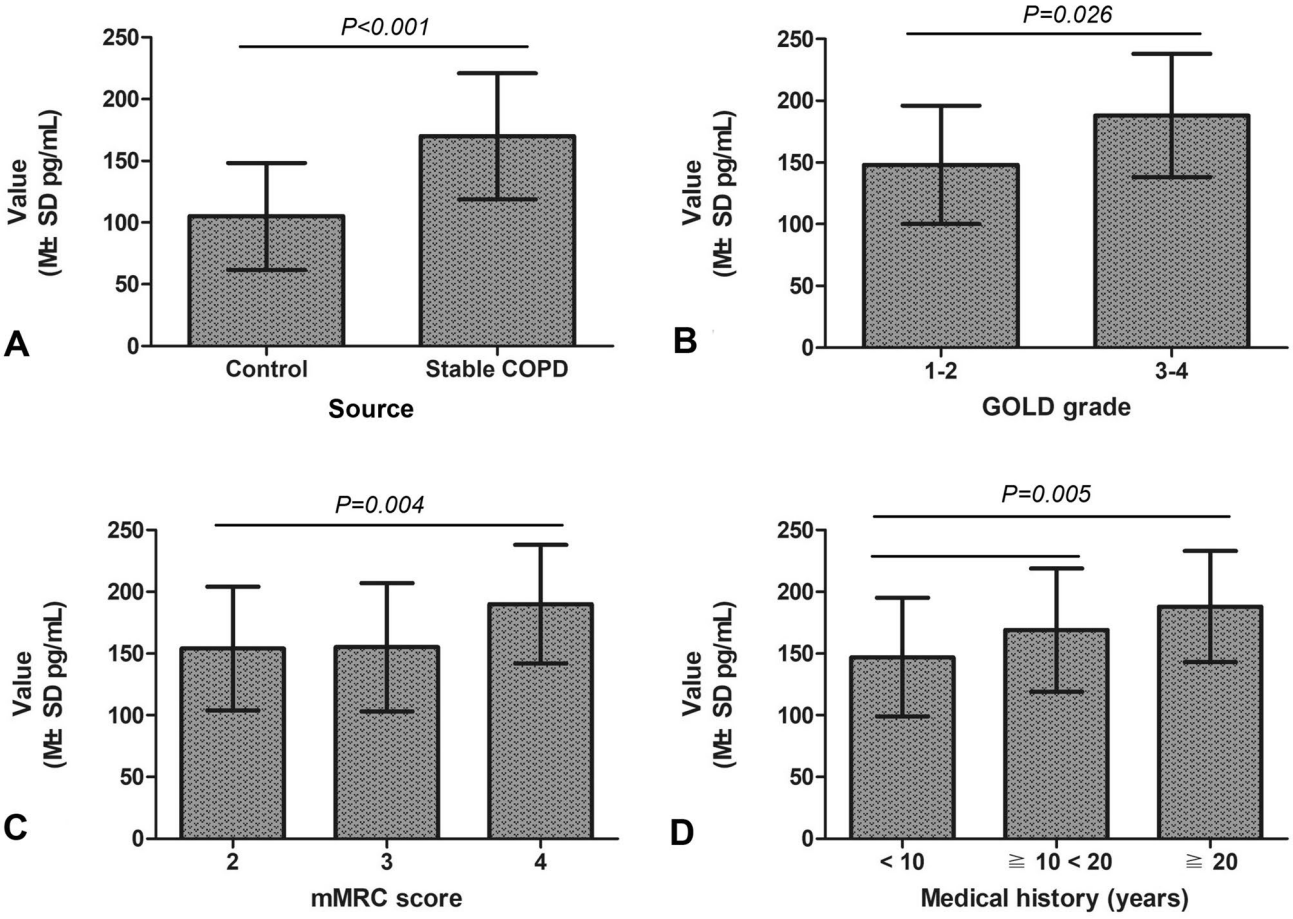
Relationship between clinical parameters and serum concentration of IL-32 in stable COPD patients. (**A**) Patients with stable COPD had a higher serum concentration of IL-32 compared with control group (*p* < 0.001). (**B**) Serum IL-32 concentration of patients with GOLD-3 and 4 was increased compared with that of GOLD-1 and 2 (*p* = 0.026). (**C**) Serum IL-32 concentration in patients with mMRC score of 4 was increased than that in patients with mMRC score of 3 and 2 (*p* = 0.004). (**D**) Serum IL-32 concentration in the patients with long clinical history was increased compared to those with short history (*p* = 0.005). COPD, chronic obstructive pulmonary disease; IL-32, interleukin-32; M ± SD, mean ± standard deviation; GOLD, Global Initiative for Chronic Obstructive Lung Disease; mMRC, modified british medical research council.

The homogeneity test of variance showed that the two population variances on the data of IL-32 concentration was equal (F = 4.005 and *p* = 0.052). The results of T test showed that serum concentration of IL-32 in patients with stable COPD (169.9 ± 51.9 pg/mL) was higher than that in healthy individuals (105.1 ± 43.3 pg/mL) (T = − 7.476, Df = 144, *p* < 0.001) (Fig. 1A; Table 1).

**Table 1:** Relationship between serum concentration of IL-32 and clinical features in stable COPD patients (n = 116).

Parameter	Group	N	Homogeneity test of variance		Serum concentration of IL-32 in stable COPD patients			
			F value	*p* value	Value (M ± SD pg/mL)	Degree of freedom	Statistical value	*p* value
Source	Control	70	4.005	0.052	105.1 ± 43.3	144	− 7.476	< 0.001
	Stable COPD	116			169.9 ± 51.9			
Gender	Male	66	0.222	0.693	166.3 ± 56.3	114	− 0.808	0.421
	Female	50			174.8 ± 52.9			
Ages	< 70	55	1.433	0.234	165.7 ± 55.2	114	− 0.703	0.484
	≧ 70	61			17,301 ± 49.6			
BMI	≧ 18.5≦ 24.9	82	1.035	0.198	169.8 ± 46.3	114	− 0.791	0.297
	≧ 25≦ 30	34			171.2 ± 51.9			
Smoking	Yes	54	1.518	0.221	176.1 ± 49.3	114	1.094	0.277
	No	62			164.6 ± 54.6			
GOLD grade	1–2	42	3.295	0.073	149.6 ± 52.3	114	− 2.254	0.026
	3–4	74			186.5 ± 50.4			
mMRC score	2	21	2.169	0.120	154.1 ± 55.3	2	5.928	0.004
	3	52			155.3 ± 52.8			
	4	43			189.8 ± 48.2^a^			
	< 10	44	0.922	0.401	147.7 ± 48.9	2	5.605	0.005
	≧ 10 < 20	33			169.9 ± 51.1^b^			
Medical history (years)	≧ 20	39			187.6 ± 45.8^b^			

*N* number, *COPD* chronic obstructive pulmonary disease, *IL-32* interleukin-32, *M* ± *SD* mean ± standard deviation, *BMI* body mass index, *GOLD* Global Initiative for Chronic Obstructive Lung Disease, *mMRC* modified british medical research council.

^a^The patients with mMRC score of 4 showed a higher concentration of serum IL-32 than those with mMRC score of 2 and 3.

^b^The concentration of serum IL-32 in the patients with a longer history was up-regulated compared to those with relatively short history.

### Serum IL-32 concentration in patients with stable COPD positively correlates with GOLD grade, mMRC score and clinical medical history

The results of the T test showed that serum IL-32 concentration in patients with stable COPD was not associated with gender, smoking and BMI (*p* > 0.05) (Table 1). The data variance test for the correlation between serum IL-32 and GOLD grade, mMRC score and clinical medical history of patients showed that the overall variance of each group was equal (F = 3.295 and *p* = 0.073 for GOLD grade; F = 2.169 and *p* = 0.120 for mMRC score; F = 0.922 and *p* = 0.401 for clinical medical history) (Table 1). The statistical results showed that serum IL-32 concentration in stable COPD patients with GOLD-3 to 4 (186.5 ± 50.4 pg/mL), mMRC score of 4 (189.8 ± 48.2 pg/mL) and longer clinical medical history (187.6 ± 45.8 pg/mL for ≧ 20 years; 169.9 ± 51.1 pg/mL for history ≧ 10 < 20 years) was higher than that in patients with GOLD-1 to 2 (149.6 ± 52.3 pg/mL) (*p* = 0.026; Fig. 1B), mMRC score of 2 (154.1 ± 55.3 pg/mL) and 3 (155.3 ± 52.8 pg/mL) (*p* = 0.004; Fig. 1C) and shorter medical history (147.7 ± 48.9 pg/mL for history < 10 years) (*p* = 0.005; Fig. 1D).

### Serum IL-32 concentration in patients with stable COPD negatively correlates with pulmonary function

**Figure 2:**
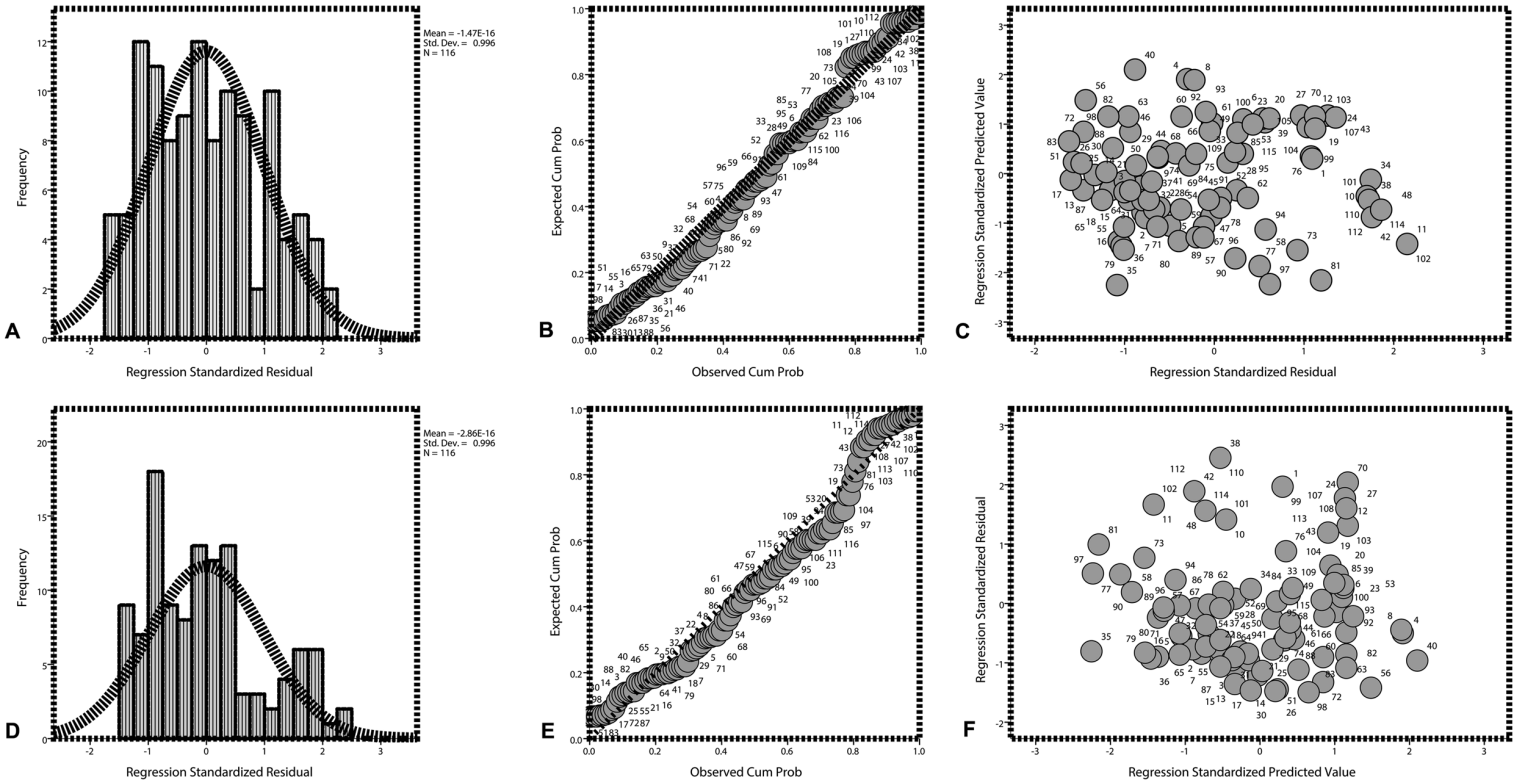
Relationship between serum IL-32 concentration and pulmonary function in stable COPD patients. (**A**) The histogram of normal curve showed that the normalized residuals were normal distributions (*p* > 0.05). (**B**) The dependent variable was approximately linear with the standardized predictive value, indicating that the IL-32 and FEV1/FVC had a negative linear correlation (*p* > 0.05). (**C**) The vast majority of normalized residuals did not exceed 3, suggesting no specific value was found. (**D**) The histogram of normal curve showed that the normalized residuals were normal distributions (*p* > 0.05). (**E**) The dependent variable was approximately linear with the standardized predictive value, indicating that the IL-32 and FEV1% predicted had a negative linear correlation (*p* > 0.05). (**F**) The vast majority of normalized residuals did not exceed 3, suggesting no specific value was found. COPD, chronic obstructive pulmonary disease; IL-32, interleukin-32; M ± SD, mean ± standard deviation; FEV1, forced expiratory volume in one second; FVC, forced vital capacity.

The serum concentration of IL-32 was negatively correlated with FEV1/FVC (*correlation coefficient* = − 0.356, *p* = 0.001; F = 11.207, *p* = 0.001; T = − 3.348, *p* = 0.001; the regression equation = *^Y* = 0.593–0.096*X*) (Fig. 2A–C; Table 2) and FEV1% predicted (*Correlation coefficient* = − 0.300, *p* = 0.001; F = 11.295, *p* = 0.001; T = − 3.361, *p* = 0.001; the regression equation = *^Y* = 0.634–0.062*X*) (Fig. 2D–F; Table 2) in stable COPD patients.

**Table 2:** Correlation between serum IL-32 concentration and pulmonary function in patients with stable COPD (N = 116).

	Items	Correlation analysis	
		IL-32 and FEV1/FVC	IL-32 and FEV1% predicted
Pearson correlation	Correlation coefficient	− 0.356	− 0.300
	*p* value	0.001	0.001
Model summary	Correlation coefficient	0.356	0.300
	R square	0.093	0.097
	Adjusted R square	0.082	0.086
	SE of the estimate	0.134	0.177
Analysis of variance	Mean square	0.203	0.355
	F value	11.207	11.295
	*p* value	0.001	0.001
Regression coefficient	Regression coefficients	0.593	0.634
	SE	0.042	0.056
	T value	− 3.348	− 3.361
	*p* value	0.001	0.001
	95% confidence interval	− 0.002% to − 0.000%	− 0.001% to − 0.000%
	Equation	*^Y* = 0.593–0.096*X*	*^Y* = 0.634–0.062*X*

*IL-32* interleukin-32, *FEV1* the value of forced expiratory volume in one second, *FVC* forced vital capacity.

### Serum concentration of IL-32 is affected by COPD grading, clinical medical history and FEV1/FVC

**Figure 3:**
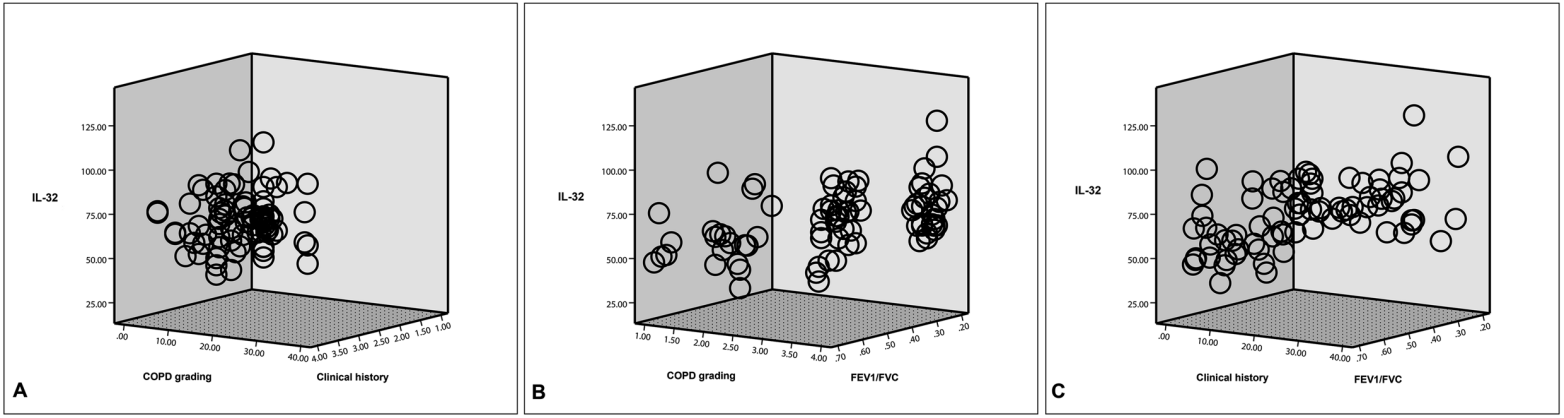
Comparison of factors affecting serum IL-32 concentration in patients with stable COPD. (**A**) The patient's COPD grading showed a more effect on IL-32 concentration than the patient's clinical history (*p* < 0.05). (**B**) The effect of FEV1/FVC on IL-32 concentration was more direct than COPD grading (*p* < 0.05). (**C**) The effect of the patient’s FEV1/FVC on IL-32 expression was greater than the medical history (*p* < 0.05). GOLD, Global Initiative for Chronic Obstructive Lung Disease; IL-32, interleukin-32; FEV1, forced expiratory volume in one second; FVC, forced vital capacity.

The stepwise regression of multiple linear showed that patient's COPD grading (*p* = 0.001; 95% CI = 6.63 to 26.61), clinical medical history (*p* = 0.023; 95% CI = 0.03 to 3.52) and FEV1/FVC (*p* = 0.001; 95% CI = − 175.16 to − 44.93) seemed to affect the serum IL-32 concentration (Fig. 3A–C; Table 3). The influence of FEV1/FVC (partial regression analysis = − 110.15) on IL-32 was greater than that of COPD grading (partial regression analysis = 16.62) and clinical medical history (partial regression analysis = 10.53).

**Table 3:** Multiple linear regression analysis to determine the factors that affect the serum concentration of IL-32 in stable COPD (N = 116).

Items	Multiple linear regression analysis for IL-32								
	Unstandardized coefficients		Variance analysis		Standardized coefficients	T value	*p* value	95% confidence interval for B	
	Partial regression analysis (B)	SE	F value	*p* value	Beta value			Lower bound	Upper bound
GOLD grading (X1)	16.62	5.03	10.86	0.001	0.29	3.29	0.001	6.63	26.61
Clinical history (X2)	10.53	2.41	6.53	< 0.001	0.21	1.853	0.023	0.03	3.52
FEV1/FVC (X3)	− 110.15	15.82	11.21	0.001	− 0.30	− 3.348	0.001	− 175.16	− 44.93
Regression equation	*^Y* = 32.1 + 16.62*X1* + 10.53*X2 *− 110.15X3								

*GOLD* Global Initiative for Chronic Obstructive Lung Disease, *IL-32* interleukin-32, *FEV1* the value of forced expiratory volume in one second, *FVC* forced vital capacity.

### Diagnostic efficacy test of serum IL-32 in distinguishing patients with COPD from healthy individuals

**Figure 4:**
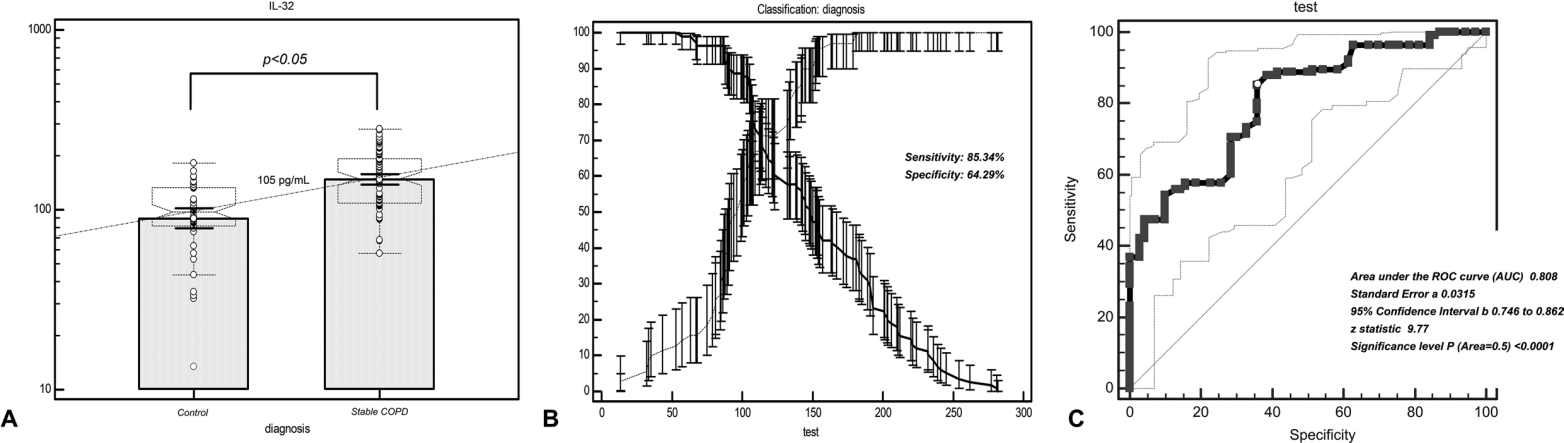
Diagnostic value and clinical significance of serum IL-32 concentration in stable COPD. (**A**) The serum IL-32 threshold value for discerning patients with COPD from healthy individuals was 105 pg/mL. (**B**) The sensitivity and specificity for distinguishing patients with COPD from healthy individuals were 85.34% and 64.29%. (**C**) The area under the curve (AUC) of serum IL-32 for discriminating COPD patients from healthy individuals was 0.808 and Z value was 9.77 (*p* < 0.001). *GOLD* Global Initiative for Chronic Obstructive Lung Disease, *AUC* area under the curve.

The ROC analysis suggested that the threshold value of serum IL-32 concentration for distinguishing patients with COPD from healthy individuals was 105 pg/mL (Fig. 4A; Table 4), the value showed a sensitivity of 85.34% and a specificity of 64.29% (Fig. 4B). Taking the value as a critical point, the area under the curve (AUC) was 0.808, the standard error was 0.0315, and the 95% confidence interval was 0.746–0.862 (Z = 9.77; *p* < 0.001) (Fig. 4C).

**Table 4:** Criterion values and coordinates of the ROC curve of serum IL-32 concentration for distinguishing COPD from healthy people.

Criterion	Sensitivity	95% CI	Specificity	95% CI	+ LR	95% CI	− LR	95% CI
> 53	100.00	96.9–100.0	12.86	6.1–23.0	1.15	0.6–2.1	0.00	–
> 63	99.14	95.3–100.0	15.71	8.1–26.4	1.18	0.7–2.0	0.055	0.008–0.4
> 87	96.55	91.4–99.1	37.14	25.9–49.5	1.54	1.1–2.1	0.093	0.03–0.2
> 90	89.66	82.6–94.5	41.43	29.8–53.8	1.53	1.2–2.0	0.25	0.1–0.4
> 94	88.79	81.6–93.9	48.57	36.4–60.8	1.73	1.3–2.2	0.23	0.1–0.4
> 101	87.93	80.6–93.2	58.57	46.2–70.2	2.12	1.7–2.6	0.21	0.1–0.4
> 105*	85.34	77.6–91.2	64.29	51.9–75.4	2.39	2.0–2.9	0.23	0.1–0.4
> 113	70.69	61.5–78.8	70.00	57.9–80.4	2.36	1.9–2.9	0.42	0.3–0.7
> 123	60.34	50.8–69.3	71.43	59.4–81.6	2.11	1.7–2.6	0.56	0.4–0.9
> 139	56.90	47.4–66.1	84.29	73.6–91.9	3.62	3.0–4.4	0.51	0.3–0.9
> 150	47.41	38.1–56.9	90.00	80.5–95.9	4.74	3.9–5.8	0.58	0.3–1.2
> 163	42.24	33.1–51.8	97.14	90.1–99.7	14.78	11.9–18.4	0.59	0.2–2.4
> 181	37.07	28.3–46.5	100.00	94.9–100.0	–	–	0.63	–

*ROC* receiver operating characteristic curve, *IL-32* interleukin-32, *COPD* chronic obstructive pulmonary disease, *95% CI* 95% confidence, +*LR* positive likelihood ratio, −*LR* negative likelihood ratio.

## Discussion

Interleukin-32 (IL-32) is originally cloned from natural killer cells by interleukin-18 (IL-18) induction, it was later discovered that its biological role is similar to cytokines and plays a key role in chronic inflammation^8,10–12^ . Previous studies show that IL-32 can induce immune cells to produce a variety of other cytokines and chemokines, involving in the specific immune response and inflammatory response of COPD^8,10,12,13^. We investigated the concentration of serum IL-32 in stable COPD patients and found that the serum concentration of IL-32 in patients with stable COPD was higher than that in healthy individuals, indicating that there was a correlation between increased serum concentration of IL-32 and COPD. Previous studies show that IL-32 can activate the phosphorylation of the nuclear transcription factor kappa B (NF-κB) and p38 mitogen-activated protein kinase (p38MAPK), inducing the production of inflammatory factors such as tumor necrosis factor-α (TNF-α), IL-1β, IL-6 and IL-8, these cytokines play an important role in the pathogenesis of COPD^10,14^.

The GOLD score of COPD patients can reflect objective severity of the disease (the higher the score, the more serious the disease). Our study showed that there was a strong positive correlation between serum IL-32 concentration and GOLD score, which suggested that IL-32 might be a molecular biomarker that reflects the severity of COPD. Previous studies show that down-regulating the concentration of IL-32 leads to the increase of some inflammatory factors, including TNF-α, IL-6, IL-8 and cell adhesion factor (ICAM)-1, this phenomenon indicates that IL-32 is an important pro-inflammatory cytokine^8,11^. The GOLD score is only an indicator of respiratory function, and there is still a certain degree of limitation for evaluating COPD as a systemic disease. The mMRC score is another indicator to evaluate the quality of life of patients with COPD. It has been widely used in the evaluation of the condition and therapeutic efficacy of patients with COPD and the prediction of the risk of death in patients^15^. Our study showed that serum IL-32 concentration in patients with stable COPD was positively correlated with mMRC score, suggesting that IL-32 was related to the severity of patients with COPD. However, the clinical evaluation of serum IL-32 to COPD still needs to be confirmed by further research. Elevated serum IL-32 concentration in patients with long clinical medical history of COPD also reflects that IL-32 could be associated with the chronic development of COPD. Because IL-32 is involved in the COPD immune response, researchers have proposed to treat COPD by inhibiting the secretion of IL-32^7^. Controlling the inflammatory response mediated by IL-32 may be another theoretical basis for the development of new drugs for the treatment of COPD in the future.

It is well known that FEV1% and FEV1/FVC are important indicators for evaluating respiratory function in patients with COPD, and the decrease in both indicates the progression of COPD and the increase risk in mortality^16^. In our study, the linear correlation analysis showed that serum IL-32 concentration in patients with stable COPD was negatively correlated with FEV1% and the FEV1/FVC, suggesting that changes in serum IL-32 can reflect the severity of COPD disease progression. The theory of airway inflammation is an important mechanism of the pathogenesis of COPD, and excessive production of inflammatory factors induces immune damage, leading to deterioration of lung function. Previous studies shows that increased IL-32 was negatively correlated with decreased FEV1, FEV1/FVC and oxygenation index in patients with acute exacerbation of COPD, confirming that IL-32 has a direct pro-inflammatory effect and also is the cause of chronic airway inflammation persisting and progressively worsening^7,17,18^. We did a multiple linear regression and found that COPD grading, clinical medical history and FEV1/FVC value in stable COPD affected the serum concentration of IL-32, and the FEV1/FVC had the most obvious influence on IL-32. As a pro-inflammatory cytokine, IL-32 not only involves in the continuous chronic inflammation of the airway but also reflects the severity of patients with COPD, and therefore it can be used as an evaluation indicator of disease progression and treatment effect in patients with COPD.

To further understand whether IL-32 has a reference value in the diagnosis of COPD, we conducted further diagnostic analysis. Through a ROC analysis, we found that the sensitivity and specificity for distinguishing patients with COPD from healthy individuals were 85.34% and 64.29%, and the area under the curve reached 0.808, indicating that IL-32 can reflect the disease occurrence of COPD and has a good value for diagnosis of COPD. It is reported that IL-32 is significantly elevated in patients with chronic inflammatory diseases of the airways, and IL-32 has the effect of inhibiting airway remodeling^7,8,10,17–19^. However, the effect of IL-32 on the inhibition of airway remodeling and the promotion of airway inflammatory response remains to be confirmed. Our findings may suggest a hypothesis that serum IL-32 may be involved in the inflammatory process of COPD, inhibition of IL-32 may cut off IL-32 mediated inflammatory response, thereby reducing airway remodeling and airflow limitation in patients with COPD, and alleviating symptoms in patients. This will be a new direction for clinical treatment of COPD.

In summary, serum IL-32 concentration is higher in patients with stable COPD than in healthy individuals, and increased IL-32 is positively correlated with GOLD grade, mMRC score and clinical medical history of patients but negatively with FEV1/FVC and FEV1% predicted, suggesting that IL-32 is involved in the chronic disease process of COPD and changes in IL-32 can be used to assess the severity of COPD and contributes to the diagnosis of COPD. However, there are also some shortcomings in this study. Firstly, the study only included Chinese patients and might have a geographical and ethnographic bias. Secondly, this study did not involve patients with acute exacerbation. Thirdly, this study did not investigate the specific intrinsic molecular mechanism of IL-32 elevation in COPD. Fourthly, this study did not involve the phenotype matter for example bronchitic vs. other phenotypes, nor did it give a definitive conclusion about the relationship between BMI and IL-32. Fifthly, there were less smokers in the control arm and non-smokers are more likely to have healthier habits like exercise and diet that may positively affect their health thus introduce a bias. In the future, the in-depth research to explain these issues should be conducted.

## Methods

### Ethics statement

This is an observational retrospective analysis (on patient’s blood specimen for test program). Informed consent was obtained from each of the recruited patients prior to entering the study in accordance with the approved ethical guidelines. The study was approved by the Research Ethics Committees of research institutes (Jining NO.1 People’s Hospital, Jining, China; Shenmu Hospital, Shenmu, China; Zhangye Second People Hospital, Zhangye, China; Minle County People’s Hospital, Minle, China; Minqin County People’s Hospital, Minqin, China; Gansu Provincial Hospital, Lanzhou, China; The First Affiliated Hospital, Xi’an Medical University, Xi’an, China).

### Objects

Between May 2015 and May 2019, a total of 116 patients from 7 hospitals (patients recruited from an indicated Ethics statement centers) were included in the observation group. At the same time, 70 outpatient health individuals distributed in the above-mentioned medical institutions were included in the control group. The demographic characteristics between the observation group and the control group, including gender, age, and smoking status, were not statistically significant (*p* > 0.05) (Table 5).

**Table 5:** Clinical information and general data of included patients (COPD = 116 cases; Control = 70 cases).

Parameters	Group	COPD (N, %)	Control (N, %)
Gender	Male	66 (56.9)	31 (44.3)
	Female	50 (43.1)	39 (55.7)
Age (years)	< 70	55 (47.4)	36 (51.4)
	≧ 70	61 (52.6)	34 (48.6)
Smoking	Yes	54 (46.6)	23 (32.6)
	No	62 (53.4)	47 (67.4)
GOLD grade	1	9 (13.6)	
	2	33 (28.4)	
	3	43 (37.1)	
	4	31 (26.7)	
	4	31 (31.6)	
mMRC score	2	21 (18.1)	
	3	52 (44.8)	
	4	43 (37.1)	
Clinical history (years)	< 10	44 (37.9)	
	≧ 10 < 20	33 (28.4)	
	≧ 20	39 (33.7)	

GOLD grade: 1 = the forced expiratory volume in one second (FEV1) % predicted is more or equal to 80%, 2 = the FEV1% predicted is more or equal to 50%, but less than 80%, 3 = the FEV1% predicted is more or equal to 30%, but less than 50%, and 4 = the FEV1% predicted is less than 30%; mMRC, modified british medical research council for dyspnea scale for symptom classification of COPD.

*COPD* chronic obstructive pulmonary disease, *GOLD* Global Initiative for Chronic Obstructive Lung Disease.

### Diagnostic criteria for patients enrolled and severity rating of COPD

The diagnosis and severity evaluation of COPD were performed by the diagnostic criteria of the Global Initiative for Chronic Obstructive Pulmonary Disease (GOLD)^1,3,6^. Objective criteria: after inhalation of 300 to 400 μg of salbutamol, the ratio of forced expiratory volume (FEV1) in the first second to forced vital capacity (FVC) is less than 0.70. The severity of COPD: GOLD-1, FEV1% predicted is more or equal to 80%; GOLD-2, the FEV1% predicted is more or equal to 50%, but less than 80%; GOLD-3, the FEV1% predicted is more or equal to 30%, but less than 50%; and GOLD-4, the FEV1% predicted is less than 30%.

### Inclusion criteria

Inclusion criteria: (1) must meet the diagnostic criteria of COPD and be a stable COPD (the patient's cough, expectoration and shortness of breath are in stable condition or just show a mild symptoms or the condition is basically restored to the state before acute exacerbation)^1,3^; (2) must have a lung function test within 3 days before enrollment; (3) no antibiotics, glucocorticoids and theophylline were used within 2 weeks before enrollment; (4) without laboratory and imaging evidences of pulmonary infection and pneumonia.

### Exclusion criteria

Exclusion criteria: (1) patients who used immunosuppressive drugs 1 week before blood collection; (2) acute infection occurred within 1 month before enrollment; (3) known etiology or pathological manifestations of airflow-limited diseases such as bronchiectasis, tuberculosis, lung cystic fibrosis and tumors; and (4) combined with serious heart, liver, kidney, hematopoietic system, endocrine system and other primary diseases, or mental illness and skin disease.

### Collection and processing of blood specimens

On the next day after the patient was enrolled, 5 mL of fasting elbow venous blood was taken in the morning, centrifuged (centrifugal radius 5 cm, rotation speed 3,000 r/min) for 10 min, and the supernatant was aspirated and stored in a − 70 °C refrigerator.

### Enzyme-linked immunosorbent assay

The concentration of IL-32 (Lanji Biological Co., Ltd., Shanghai, China) in serum was measured by sandwich-type ELISA according to the method provided by the kit: (1) added sample dilution of 100 μL on test samples and standard samples, and incubated for 2 h at room temperature; (2) after washing, added test antibody of 100 μL and incubated for 2 h at 37 °C; (3) after washing, added streptavidin-HRP working solution of 100 μL and incubated at room temperature for 20 min in the dark; (4) after washing, added substrate solution of 100 μL and incubated at room temperature for 20 min in the dark; (5) added stop solution of 50 μL and thoroughly mixed; (6) the optical density of each well was measured with a microplate reader set at 450 nm.

### Modified British Medical Research Council (mMRC) score

Dyspnea of patients was evaluated by the modified british medical research council (mMRC) scale that divided into 5 points^15^. 0 points: dyspnea will only occur after excessive activity; 1 point: dyspnea will occur after rapid walking; 2 points: dyspnea is more likely to occur during normal walking than normal people; 3 points: need to stop and breath after walking about a few minutes on even ground; 4 points: obvious breathing difficulty that affects normal life and work.

### Objective evaluation

All included patients must undergo three lung function tests before collecting the specimens and cannot be performed on the same day (three days before including into the study, the lung function test was performed once a day). The average of the three tests was calculated as statistical data. This study selected two data directly related to the diagnosis and classification of COPD: FEV1/FVC and FEV1% predicted.

### Statement for strengthening the reporting of observational studies in epidemiology (STROBE)

This study was a retrospective case–control study and was divided into seven sections: headings, abstracts, background presentations, methods, results, discussion, and other information. The study clearly described the grouping of the study and the sample size, study design, and test indicators. The statistical methods were described in detail, and the systematic errors and data offsets of the variables were analyzed. The data of the study were detailed in the results section, and the main findings of the study were explained in discussion. The keywords and terminology used in this study help to ensure the correct indexing of article in the electronic database.

### Statistical processing of research data

The statistical software used in this study included IBM SPSS Statistics and MedCalc statistical software. The count data was expressed in terms of rate and analyzed by chi-square test and Fisher exact probability method. The measurement data was expressed in the form of mean ± standard deviation, and the comparison between groups was performed by analysis of variance and T test. The receiver operating characteristic (ROC) curve was used to determine the threshold of observed indicator and evaluate the diagnostic efficacy. Levene's Test for Equality of Variances was performed to determine whether the data is from a normal distribution or not. A statistical *p* value of less than 0.05 was considered to be statistically significant.

## Abbreviations

BMI: Body mass index
CI: Confidence interval
COPD: Chronic obstructive pulmonary disease
FEV1: The value of forced expiratory volume in one second
FVC: Forced vital capacity
GOLD: The Global Initiative for Chronic Obstructive Pulmonary Disease
ICAM: Cell adhesion factor
IL-1β: Interleukin 1β
IL-6: Interleukin 6
IL-8: Interleukin 8
IL-32: Interleukin 32
IL-18: Interleukin 18
NF-κB: Nuclear transcription factor kappa B
mMRC: The modified british medical research council
p38MAPK: P38 mitogen-activated protein kinase
ROC: Receiver operating characteristic
STROBE: Statement for strengthening the reporting of observational studies in epidemiology
TNF-α: Tumor necrosis factor-α
